# Interaction of Ferroptosis and Immune-Mediated Inflammation in Psoriasis

**DOI:** 10.3390/antiox15030382

**Published:** 2026-03-18

**Authors:** Emanuele Giorgio, Cristiana Galeano, Giuseppe Natali, Lavinia Petriaggi, Maria Concetta Faniello, Elzbieta Janda, Francesco Saverio Costanzo, Anna Martina Battaglia, Flavia Biamonte

**Affiliations:** 1 Department of Experimental and Clinical Medicine, Magna Graecia University of Catanzaro, 88100 Catanzaro, Italy; emanuele.giorgio@unicz.it (E.G.); cristiana.galeano@studenti.unicz.it (C.G.); lavinia.petriaggi@unicz.it (L.P.); faniello@unicz.it (M.C.F.); fsc@unicz.it (F.S.C.); 2 Department of Health Sciences, Magna Graecia University of Catanzaro, 88100 Catanzaro, Italy; giuseppe.natali@studenti.unicz.it (G.N.); janda@unicz.it (E.J.)

**Keywords:** psoriasis, ferroptosis, oxidative stress, iron metabolism, keratinocytes, immune-epidermal crosstalk

## Abstract

Psoriasis is classically defined as an immune-mediated disease. However, many patients do not achieve durable remission after immune-targeted therapies, suggesting that further pathogenic mechanisms may contribute to the persistence of psoriasis. Here, we propose ferroptosis, an iron-dependent regulated cell death driven by lipid peroxidation and failure of lipid repair, as a potential link between metabolic stress and immune-mediated inflammation in psoriasis. We summarize experimental evidence showing that membrane lipids remodeling, antioxidant suppression, lipid peroxidation, and dysregulated iron handling together define ferroptosis-permissive niches within psoriatic lesions. We also discuss functional studies demonstrating that ferroptosis modulation can reshape psoriasiform inflammation and explore how ferroptotic stress may amplify inflammatory signaling at the immune-epidermal interface, reinforcing IL-17/TNF/IFN-γ pathways. Finally, we discuss ferroptosis-related transcriptomic signatures as a potential approach to stratify psoriasis, capturing metabolic features that are not reflected by cytokine profiling. The translational opportunities and constraints for ferroptosis-targeted interventions are outlined, highlighting epidermal redox homeostasis as a new therapeutic frontier in psoriasis.

## 1. Introduction

Psoriasis is a chronic, immune-mediated inflammatory disease affecting more than 125 million individuals worldwide, imposing a substantial burden on quality of life and healthcare systems [1,2,3]. Plaque psoriasis is characterized by the development of a well-demarcated, erythematous, scaly lesion that reflects profound alterations in epidermal architecture and function [4,5,6]. Histologically, psoriatic plaques display marked epidermal hyperplasia (acanthosis), elongated rete ridges, parakeratosis, and a severely shortened keratinocyte turnover time, with differentiation becoming incomplete and spatially disorganized [7,8]. This aberrant program is accompanied by loss of barrier integrity, altered lipid composition, and increased trans epidermal water [9,10]. Within plaques, the keratinocytes acquire a pro-inflammatory phenotype, producing cytokines, chemokines and antimicrobial peptides that sustain immune cell recruitment and activation [11,12,13]. Thus, the psoriatic epidermis is not merely a passive target of inflammation but an active driver of disease, in which dysregulated proliferation, differentiation, and metabolism converge to perpetuate the chronic cutaneous inflammation.

Current pathogenic models define psoriasis as the product of a self-amplifying dialog between the immune system and the epidermis, in which genetically primed keratinocytes aberrantly respond to environmental and inflammatory cues, fueling a pathogenic interleukin-23 (IL-23)/interleukin-17 (IL-17)/tumor necrosis factor (TNF) axis that, in turn, sustains chronic inflammation and epidermal hyperplasia [14,15]. Despite the remarkable clinical success of cytokine-targeted therapies, a substantial fraction of patients remains only partially responsive or refractory, and disease frequently relapses upon treatment discontinuation. These limitations indicate that immune dysregulation alone cannot fully explain disease persistence, heterogeneity, and chronicity [15,16].

Psoriatic epidermis is characterized by profound alterations in lipid composition and heightened oxidative stress [17]. This perspective raises an interesting question: could ferroptosis, a new form of regulated cell death driven by iron-dependent lipid peroxidation and collapse of antioxidant defenses [18,19], constitute a mechanistic link between epidermal metabolic stress and immune activation in psoriasis? This is plausible. Ferroptotic cells release oxidized lipids and danger-associated molecular patterns (DAMPs) capable of activating innate immune response and reshaping tissue microenvironments [20]. Furthermore, in immune-mediated diseases, ferroptosis is implicated in macrophage polarization, cytokine amplification, and tissue damage, as demonstrated in arthritis and autoimmunity models [21,22]. Indeed, over the last few years, transcriptomic and biochemical analyses have revealed downregulation of glutathione peroxidase 4 (*GPX4*), accumulation of lipid peroxidation products, and dysregulation of iron-handling genes in human psoriatic lesions [18,23,24]. Furthermore, pharmacological or genetic inhibition of ferroptosis in keratinocytes attenuates imiquimod (IMQ)-induced psoriasiform inflammation [25].

In this review, we first describe the molecular features of ferroptosis in the epidermal context and then discuss human and experimental evidence linking ferroptotic stress to psoriatic inflammation. We also discuss how lipid and iron metabolism rewire keratinocyte vulnerability, how ferroptosis interfaces with immune-mediated responses, and how ferroptosis-related gene signatures enable psoriatic patient stratification. Finally, we explore the translational implications of targeting ferroptosis in psoriasis, proposing that modulation of redox homeostasis in keratinocytes may complement immunotherapy and open new therapeutic horizons. Through this lens, psoriasis emerges not only as an immune-mediated disease, but as a redox–metabolic–immune disorder in which ferroptosis may constitute a central pathogenic axis.

## 2. The Molecular Basis of Ferroptosis

Ferroptosis is a regulated form of cell death driven by the iron-dependent accumulation of lipid hydroperoxides within cellular membranes [26,27,28,29,30]. Ferroptosis is distinguished from other regulated cell death by its strict dependence on three fundamental conditions: (i) the intracellular iron availability, (ii) the enrichment of oxidizable polyunsaturated fatty acid (PUFA)-containing phospholipids, and (iii) the failure of lipid peroxide detoxification systems. The concomitant absence of caspase activation and mixed lineage kinase domain-like pseudokinase (MLKL) phosphorylation provides a robust fingerprint separating ferroptosis from apoptosis and necroptosis [31,32,33].

Iron is indispensable for cellular metabolism, yet potentially lethal when present in excess. The ferroptotic cascade is initiated by an expansion of the cytosolic labile iron pool (LIP), which fuels Fenton and Haber–Weiss reactions, generating highly reactive hydroxyl radicals. These radicals catalyze the oxidation of membrane lipids, particularly PUFA-containing phospholipids [34,35,36,37,38]. Cellular iron homeostasis is governed by a tightly regulated network involving transferrin receptor (TFRC)-mediated uptake, ferritin (FTH1/FTL)-based storage, and ferroportin (FPN)-dependent export [39,39,40,41,42,43]. Perturbations of this network, whether through increased iron import, impaired sequestration, or enhanced ferritinophagy, shift cells toward a pro-oxidant state permissive for ferroptosis [44,45].

Ferroptosis is driven by the peroxidation of PUFA-containing phospholipids within cellular membranes. This process is enzymatically primed by Acyl-CoA synthetase long-chain family member 4 (ACSL4), which selectively activates PUFAs such as arachidonic and adrenic acid, while lysophosphatidylcholine acyltransferase 3 (LPCAT3) incorporates them into membrane phospholipids. The accumulation of lipid hydroperoxides destabilizes membrane architecture, alters biophysical properties, and ultimately leads to catastrophic membrane failure [46,47]. Thus, ferroptosis is not merely an oxidative event, but the endpoint of a lipid remodeling program that determines which cells become permissive to death. This pro-oxidant process is counteracted by a network of membrane-protective systems centered on GPX4, a selenoprotein uniquely able of reducing phospholipid hydroperoxides to their corresponding alcohols within membranes [48]. GPX4 activity is critically dependent on reduced GSH, whose synthesis requires cysteine availability through the system xc- antiporter (SLC7A11/SLC3A2) [48,49]. Depletion of cysteine, inhibition of GSH synthesis, or direct inactivation of GPX4 leads to unchecked lipid peroxidation and rapid ferroptotic collapse [50]. *GPX4* deletion causes embryonic lethality, and its loss in epithelial tissues leads to severe barrier defects, underscoring its essential role in preserving membrane integrity [51,52]. Beyond GPX4, ferroptosis suppressor protein 1 (FSP1, formerly AIFM2) operates independently of GSH by reducing coenzyme Q10 (CoQ10) to its lipophilic antioxidant form, ubiquinol, thereby intercepting lipid radical propagation within membranes [53,54]. Additional protective layers include the GTP cyclohydrolase 1 (GCH1)-tetrahydrobiopterin (BH4) axis and endosomal sorting complex required for transport (ESCRT) complex III (ESCRT-III)-mediated membrane repair. These systems allow cells to buffer transient oxidative insults and attenuate ferroptosis susceptibility [55,56].

## 3. The Emerging Role of Ferroptosis in Psoriasis Pathogenesis

**Figure 1:**
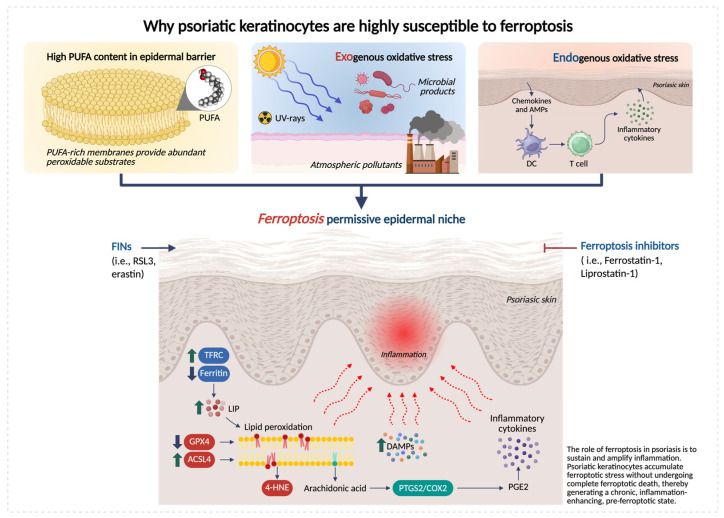
Keratinocyte-intrinsic determinants of ferroptosis susceptibility in psoriatic skin. Schematic representation of the epidermal metabolic and redox alterations that make psoriatic keratinocytes highly susceptible to ferroptosis. Psoriatic keratinocytes exhibit three major vulnerability factors: (i) high PUFA content, supplying abundant substrates for lipid peroxidation; (ii) exogenous oxidative stress from ultraviolet (UV) radiation, atmospheric pollutants and microbial stimuli; and (iii) endogenous oxidative stress driven by elevated inflammatory cytokines. These inputs converge to impair antioxidant defenses (reduced GPX4 and GSH), increase ACSL4-dependent PUFA enrichment, and disrupt iron homeostasis, collectively promoting excessive lipid peroxidation and accumulation of toxic species, such as 4-hydroxynonenal (4-HNE) and oxidized phospholipids (OxPLs). Together, these alterations create a ferroptosis-permissive epidermal niche that lowers the threshold for lipid-driven injury and amplifies psoriatic inflammation. Created in BioRender. Biamonte, F. (2026) https://BioRender.com/7q05nh6.

Over the past 5 years, multiple lines of evidence highlight that psoriatic lesion exhibits (i) impaired membrane lipid peroxide detoxification system (GPX4/GSH axis), (ii) increased availability of peroxidizable substrates via lipid remodeling/trafficking (e.g., ACSL4), and (iii) inflammatory iron flux and oxidative pressure, together creating ferroptosis-permissive epidermal niches [19,25] (Figure 1).

Epidermal homeostasis is intrinsically dependent on lipid metabolism. The epidermis is rich in phospholipids containing PUFAs, which are important for barrier formation, cell–cell cohesion, and signaling processes [57]. During terminal differentiation, keratinocytes undergo extensive membrane biogenesis and lipid remodeling to form the cornified envelope [58,59]. As basal cells exit the cell cycle and migrate to suprabasal layers, the composition of fatty acids and membrane phospholipids changes. This process can increase the incorporation of PUFAs into cellular membranes, which may transiently enhance susceptibility to lipid peroxidation and ferroptotic stress [60]. Because membrane lipids are continuously remodeled, keratinocytes rely on protective systems that preserve phospholipid integrity. Indeed, *GPX4* deletion in keratinocytes causes rapid epidermal degeneration and catastrophic barrier failure [61]. Concomitantly, keratinocytes are chronically exposed to both exogenous and endogenous sources of oxidative stress. UV radiation, atmospheric pollutants, and microbial products converge on the epidermal surface, while inflammatory cytokines and infiltrating immune cells generate sustained intracellular reactive oxygen species (ROS) [62].

Ferroptotic stress may play a dual role in psoriasis, promoting inflammation while, in certain contexts, limiting keratinocyte hyperproliferation. The type and strength of the evidence depend on whether observations derive from human lesions, keratinocyte-based experiments, or in vivo models. Shou et al. demonstrated that lipid oxidation in keratinocytes is strongly associated with the activation of Th17/Th22-related inflammatory signatures [25]. Later, Zhou et al. (2022) found that ferroptotic insults can cause the release of DAMPs/alarmins and engage eicosanoid pathways via prostaglandin endoperoxide synthase 2 (PTGS2)/cyclooxygenase-2 (COX-2), potentially coupling arachidonic acid metabolism to cytokine amplification [19]. In 2022, Liu et al. demonstrated that in psoriatic lesions, ACSL4 expression is increased and positively correlates with inflammatory mediators (e.g., TNF, IL-6, IL-8, IL-17A) and psoriasis area severity index (PASI) score. Conversely, *ACSL4* inhibition reduced both ferroptotic activation and cytokine induction. Interestingly, they also showed that erastin promotes the expression of inflammatory cytokines in keratinocytes, while ferrostatin-1 (Fer-1) dampen these outputs [63].

Not all mechanistic data point in the same direction. Wu et al. (2024) demonstrated that GPX4 inhibitor RAS-selective lethal 3 (RSL3) in HaCaT keratinocytes reduced proliferation, thus proposing ferroptosis induction as a protective mechanism in psoriasis vulgaris [23]. In psoriasis-like in vitro and in vivo settings, RSL3 reduced the hyperproliferation marker keratin 6 (*KRT6*) and increased differentiation markers, including filaggrin (*FLG*). Inflammatory chemokines were also largely reduced in vitro, raising the possibility that within certain “physiological windows,” ferroptotic stress may restrain hyperproliferation and partially normalize differentiation other than fostering inflammation, potentially via ROS-triggered antioxidant programs (e.g., nuclear factor erythroid 2-related factor 2, NRF2) [23]. These apparently divergent results converge toward a unified model in which the biological impact of ferroptosis in psoriatic skin depends on the intensity, duration, and contextual integration of lipid peroxidation stress. High and sustained ferroptotic pressure, particularly in the presence of cytokine-driven oxidative load and abundant PUFA-containing phospholipids, favors inflammatory amplification. Conversely, sublethal or transient ferroptotic cues may restrain keratinocyte hyperproliferation and support partial restoration of differentiation programs. Within this continuum, the dual nature of ferroptosis emerges not as a contradiction but as a context-dependent spectrum shaped by microenvironmental cues, redox balance, and the differentiation state of keratinocytes.

In vivo data further highlight the functional relevance of ferroptosis in the psoriasis. Shou et al. demonstrated that pharmacological ferroptosis blockade using Fer-1 mitigated IMQ-induced psoriasiform dermatitis [25]. Zhou et al. emphasized that ferroptosis inhibitors (Fer-1, liproxstatin-1) act as radical-trapping antioxidants that can modulate inflammation in keratinocytes and psoriasiform in vivo models [19]. More direct support comes from genetic models. Vats et al. (2024) provided direct proof-of-principle that ferroptosis confined to a minority of keratinocytes can initiate and sustain psoriasis-like, systemic inflammation [64]. In the K14/Gpx4 model, sporadic phospholipid peroxidation in a subset of basal keratinocytes recapitulates key features of psoriasis. They propose that ferroptosis generates oxidized free fatty acids and oxidized phospholipids (OxPLs), including oxidized phosphatidylethanolamine (oxPE) species with signaling activity, which promote proliferation and impair differentiation in neighboring keratinocytes, hereby explaining the paradoxical coexistence of cell death and epidermal hyperplasia. They further hypothesize that oxidized lipid species and adducts may be taken up by antigen-presenting cells, potentially promoting lymphocyte Th1 and IL-23/Th17 responses and establishing a feed-forward epithelial–immune loop that maintains disease [64,65].

Transcriptomic analyses reinforce this scenario. Wu et al. (2023) [66] performed multi-dataset analyses and identified a set of differentially expressed ferroptosis-related genes (DE-FRGs) able of separating psoriasis from healthy skin, including genes tied to redox control, mitochondrial function, and lipid metabolism (e.g., phosphatidylethanolamine binding protein 1 (*PEBP1*), protein kinase AMP-activated catalytic subunit alpha 2 (*PRKAA2*), acyl-CoA synthetase family member 2 (*ACSF2*), among others). Their pathway analyses connected these markers to innate immune sensing networks (nod-like receptor (NLR), toll-like receptor (TLR), retinoic acid-inducible gene-I (RIG-I)-like receptor (RLR) pathways) and immune cell infiltration patterns consistent with established psoriasis immunobiology [66]. While these results are inherently associative and limited by bulk-tissue averaging, they support the idea that ferroptosis-related transcriptional states align with psoriatic immune activation and may be exploitable for biomarker development and stratification.

## 4. Ferroptosis as a Driver of Immune-Epidermal Crosstalk in Psoriasis

**Figure 2:**
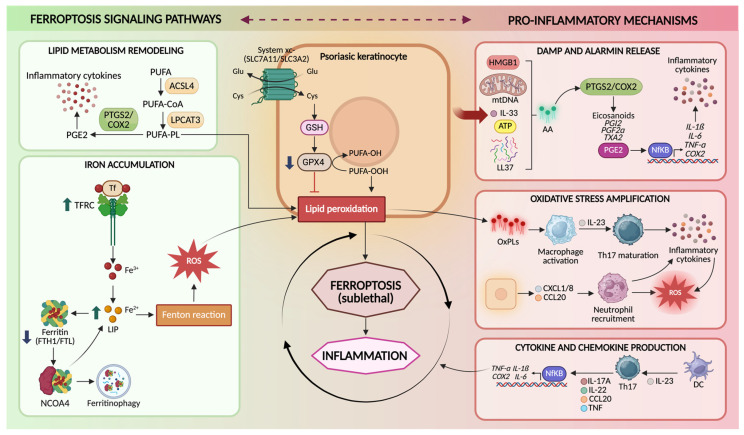
Ferroptosis signaling pathways in psoriatic keratinocytes. Schematic representation of the molecular mechanisms linking ferroptosis to psoriasis in keratinocytes. Ferroptosis is promoted by lipid metabolism remodeling, in which ACSL4 and LPCAT3 incorporate PUFAs into membrane phospholipids that become susceptible to peroxidation, and by iron accumulation, driven by increased TFRC-mediated iron uptake and nuclear receptor coactivator 4 (NCOA4)-dependent ferritinophagy, which expands the LIP and promotes ROS generation through the Fenton reaction. The system Xc^−^–GSH–GPX4 axis normally protects cells from ferroptosis by importing cystine for glutathione (GSH) synthesis and enabling GPX4 to detoxify lipid hydroperoxides. Impairment of this antioxidant system leads to lipid peroxidation and sublethal ferroptotic stress in keratinocytes. Ferroptosis-associated lipid oxidation promotes the release of DAMPs and alarmins–such as high mobility group box 1 (HMGB1), mitochondrial DNA (mtDNA), interleukin-33 (IL-33), adenosine triphosphate (ATP), cathelicidin antimicrobial peptide LL-37 (LL-37)-activation of NF-κB, and COX-2-dependent eicosanoid production. These signals amplify oxidative stress, stimulate macrophage activation and IL-23-driven Th17 responses, promote neutrophil recruitment through chemokines-such as C-X-C motif chemokine ligand 1/8 (CXCL1/8), C-C motif chemokine ligand 20 (CCL20)-and enhance pro-inflammatory cytokine production (IL-17A, IL-22, TNF-α, IL-1β, IL-6), contributing to psoriasis pathogenesis. Created in BioRender. Biamonte, F. (2026) https://BioRender.com/7q05nh6.

Psoriasis is sustained by the crosstalk between keratinocytes and immune cells, in which epidermal stress responses and cytokine-driven inflammation reinforce each other [13]. In this scenario, ferroptosis is best understood not only as a terminal cell death program but as a microenvironmental event able of reshaping immune–epidermal communication [64]. The keratinocytes undergoing ferroptotic stress (and, in some contexts, ferroptotic death) generate OxPLs, reactive aldehyde adducts, eicosanoid intermediates, and danger-associated signals, that can engage innate sensing pathways, modulate antigen presentation, and tune cytokine networks [67]. The result is a model in which ferroptosis functions as a “signals generator,” converting metabolic redox imbalance into immunological amplification (Figure 2).

### 4.1. Ferroptotic Stress in Keratinocytes as a Source of Inflammatory Signaling

Keratinocytes are recognized as active immune effector cells. In psoriasis, they produce cytokines (e.g., IL-1 family members), chemokines (e.g., CXCL1/8, CCL20), antimicrobial peptides, and lipid mediators that recruit and instruct leukocytes [68]. Ferroptosis adds a mechanistically distinct layer to this effector capacity by altering the quality and persistence of epidermal danger signaling. Unlike apoptosis, which is typically immunologically silent, ferroptosis is characterized by extensive membrane lipid oxidation and secondary formation of bioactive lipid species [69]. These products can directly stimulate inflammation, amplify cytokine responsiveness, and potentially generate neo-antigenic lipid-protein adducts [25,70]. Conceptually, ferroptotic keratinocytes are not merely dying cells; they are metabolically rewired cells that, prior to or during death, shift toward an inflammatory secretory state under redox pressure [71]. This is particularly plausible in psoriasis because keratinocytes already exist in a cytokine-saturated environment and are primed to translate stress into inflammatory transcriptional programs [72]. Ferroptotic stress thus increases the probability that keratinocyte-derived signals reach the threshold required to sustain leukocyte recruitment and activation. Furthermore, oxidized phospholipids and lysophospholipid derivatives generated during ferroptosis may diffuse locally and act as paracrine mediators, inducing proliferative responses, dysregulation of differentiation, and stress signaling in adjacent keratinocytes [70]. This offers a mechanistic explanation for a classic psoriasis paradox: the coexistence of keratinocyte death signals with marked epidermal hyperproliferation. Rather than being mutually exclusive, localized ferroptosis could generate lipid mediators that stimulate compensatory proliferation and aberrant differentiation in surrounding epidermal compartments, thereby reinforcing plaque architecture [64,73].

### 4.2. Oxidized Lipids as DAMP-like Mediators and Inflammatory Signaling Molecules

A defining feature of ferroptosis is the accumulation of oxidized PUFA-phospholipids and their downstream breakdown products. These molecules are not inert biomarkers; many of them have intrinsic signaling properties and can function in a DAMP-like manner [74]. Two classes are especially relevant to psoriasis: (i) OxPLs and hydroperoxy-PE species, and (ii) reactive aldehydes and protein adducts (e.g., 4-HNE) [75,76,77]. OxPLs can be recognized by pattern recognition mechanisms and can modulate dendritic cell and macrophage activation states. Importantly, OxPLs can also be internalized by antigen-presenting cells, potentially contributing to antigenic diversification [70]. In keratinocyte-rich tissues, lipid oxidation products may therefore function as a bridge between metabolic injury and immune priming. In addition to that, lipid peroxidation generates electrophilic aldehydes such as 4-HNE that covalently modify proteins, altering function and potentially generating neo-epitopes [78]. In psoriasis, elevated 4-HNE has been linked to ferroptosis-associated states and is mechanistically positioned to both propagate ferroptosis (by impairing pro-survival pathways) and promote inflammation (by activating stress kinases and COX-2–linked eicosanoid programs) [79]. These adducts may also be handled by antigen-presenting cells, enabling a route through which ferroptosis could contribute to adaptive immune activation beyond generic DAMP release [80]. A major amplification route connecting lipid peroxidation to inflammation is the eicosanoid axis PTGS2/COX-2, which is often induced during ferroptotic stress and can accelerate conversion of arachidonic acid into inflammatory lipid mediators. While COX-2 induction is not exclusive to ferroptosis, its integration with iron-driven lipid oxidation provides a plausible mechanism by which ferroptotic stress biases the epidermal lipid mediator landscape toward inflammation and leukocyte recruitment [81].

### 4.3. Cytokine Loops as Both Drivers and Consequences of Ferroptotic Stress

Psoriasis is sustained by cytokine feedback loops involving IL-17A/F, TNF, and interferon-gamma (IFN-γ), which together maintain inflammatory state on keratinocytes [82]. Ferroptosis intersects these loops in bidirectional ways. (i) Cytokines prime ferroptotic permissiveness: TNF and IFN-γ can induce oxidative stress and remodel metabolism in keratinocytes, increasing ROS production and altering lipid metabolic programs [76]. In parallel, chronic cytokine exposure can impair the GPX4-GSH axis by limiting cysteine availability, altering GSH metabolism, and imposing endoplasmic reticulum and mitochondrial stress [83]. The net effect is to lower the ferroptotic threshold: membranes become more oxidizable and less repairable. (ii) Ferroptotic stress amplifies cytokine release and responsiveness: once lipid peroxidation proceeds, keratinocytes can shift toward enhanced expression of inflammatory mediators and chemokines. Lipid oxidation products and aldehyde stress activate transcriptional regulators (e.g., nuclear factor kappa B (NF-κB)-linked programs) and can increase sensitivity to cytokine signaling [79,84]. Thus, a keratinocyte undergoing ferroptotic stress becomes more responsive to IL-17/TNF/IFN-γ, producing more chemokines, antimicrobial peptides, and inflammatory lipid mediators, further recruiting immune cells that sustain the cytokine milieu [25]. This creates a self-reinforcing circuit: cytokines raise ferroptotic pressure; ferroptotic stress raises inflammatory response and cytokine sensitivity. Importantly, sublethal or “pre-lethal” ferroptotic stress states, characterized by lipid peroxide accumulation and redox imbalance, may be sufficient to alter keratinocyte immune function and maintain inflammation.

### 4.4. Ferroptosis and Its Crosstalk with Macrophages, Neutrophils, and Th17 Immunity

In psoriasis, macrophages, neutrophils, dendritic cells, and Th17/Th1 lymphocytes form an interdependent network with keratinocytes [85]. Ferroptotic keratinocytes can shape this network through multiple routes. (i) Oxidized lipids can influence macrophages activation and polarization [86]. In the psoriatic dermis, macrophages are exposed to keratinocyte-derived OxPLs, aldehyde adducts, and cytokines, which may bias them toward pro-inflammatory phenotypes and enhance production of TNF, IL-1, and IL-23. In parallel, macrophages act as scavengers for oxidized material; inefficient clearance could prolong antigenic and inflammatory exposure, whereas efficient efferocytosis-like programs could limit it [87]. Given that ferroptosis may generate debris with distinct immunogenicity, macrophage handling of ferroptotic keratinocytes may be a critical determinant of whether ferroptosis resolves or sustains inflammation [88]. (ii) Neutrophil infiltration is a hallmark of psoriasis [89]. Keratinocyte-derived chemokines represent a key node for neutrophil recruitment, and ferroptotic stress may amplify their production. Neutrophils, in turn, generate ROS and release inflammatory mediators and enzymes that intensify oxidative pressure within plaques, potentially accelerating lipid peroxidation in keratinocytes [90]. This creates a redox escalation loop: ferroptotic stress recruits’ neutrophils while neutrophil oxidative burst increases ferroptotic pressure. Furthermore, neutrophil-derived lipid mediators and oxidative enzymes could modify the local lipid oxidation landscape, shaping the pool of OxPLs available for immune recognition and signaling. (iii) The IL-23/Th17 pathway is central in psoriasis. Ferroptotic keratinocytes may contribute to Th17 skewing both indirectly and directly. Indirectly, ferroptotic stress increases keratinocyte production of cytokines and chemokines that recruit and activate dendritic cells and macrophages, promoting IL-23 production and Th17 maintenance. Directly, OxPLs and lipid-protein adducts derived from ferroptosis may be taken up by antigen-presenting cells and contribute to antigenic landscapes that support T cell activation [91].

**Figure 3:**
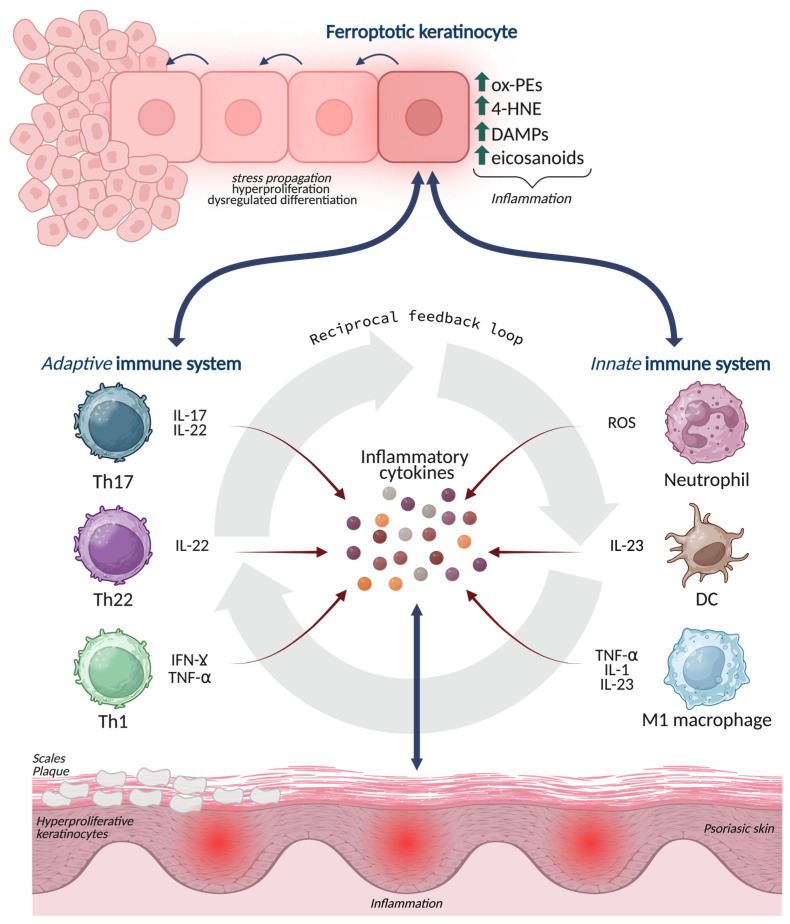
Ferroptosis-driven inflammatory signaling at the immune-epidermal interface in psoriasis. Ferroptotic stress in epidermal keratinocytes leads to the accumulation of oxidized phospholipids, 4-HNE adducts, ROS, and eicosanoid intermediates, which act as danger signals and amplify inflammation. These lipid-based mediators modulate crosstalk with innate (neutrophils, M1 macrophages, dendritic cells) and adaptive (Th17, Th22, Th1) immune cells, promoting the release of IL-17, IL-22, IFN-γ, TNF-α, and IL-23. The figure depicts how ferroptosis enhances immune recruitment, cytokine output, and redox imbalance, establishing a self-reinforcing loop that sustains keratinocyte hyperproliferation, abnormal differentiation, and chronic psoriatic inflammation. Created in BioRender. Biamonte, F. (2026) https://BioRender.com/7q05nh6.

Taken together, these findings support an integrated circuit in which ferroptosis sits at the center of immune-epidermal crosstalk (Figure 3). This new biological concept predicts that patients (or plaque regions) with stronger lipid peroxidation, altered iron handling, and weaker GPX4 defenses will exhibit more robust myeloid/Th17 activation. It also suggests that therapies targeting ferroptosis, either by restoring lipid repair capacity, limiting PUFA peroxidation, or modulating iron flux, could complement cytokine blockade by disrupting the epidermal source of inflammatory reinforcement rather than only neutralizing downstream immune mediators.

## 5. Ferroptosis-Associated Gene Signatures and Patient Stratification in Psoriasis

Recently, multiple bioinformatic analyses have identified FRG programs that distinguish psoriatic lesions from healthy skin with high fidelity [66,92]. These signatures integrate genes governing GSH metabolism (e.g., glutamate-cysteine ligase catalytic subunit (*GCLC*), glutathione-specific gamma-glutamylcyclotransferase 1 *CHAC1*), lipid remodeling and trafficking (e.g., *ACSF2*, *PEBP1*), mitochondrial and redox control (e.g., CDGSH iron sulfur domain 1 (*CISD1*), translocase of inner mitochondrial membrane 9 (*TIMM9*)), and stress-response regulators (e.g., *PRKAA2*, tribbles pseudokinase 2 (*TRIB2*), mouse double minute 2 (*MDM2*)). Importantly, these FRGs do not operate as isolate actors: functional enrichment analysis linked them to converging biological processes, such as innate immune sensing networks (TLR, NLR, RIG-I–like receptors), cytokine signaling, and epidermal differentiation programs [66].

Clustering approaches based on FRG expression have revealed different molecular subtypes of psoriasis [93]. In these analyses, one cluster typically exhibits high expression of pro-ferroptotic and redox-stress genes (e.g., elevated *CHAC1*, altered GSH pathways, lipid metabolic skewing), accompanied by enrichment of pathways related to programmed cell death, negative regulation of epithelial proliferation, and stress signaling [93]. This “ferroptosis-high” state correlates with more severe inflammatory programs and poorer predicted prognosis [93,94]. A second cluster displays a comparatively restrained ferroptotic profile, with enrichment of metabolic buffering and differentiation-related pathways and a more favorable disease phenotype. Immune deconvolution analyses further demonstrate that ferroptosis-defined subtypes profoundly differ in their cellular microenvironment [93,94]. “Ferroptosis-high” plaques show increased infiltration of activated CD4^+^ T cells, neutrophils, regulatory T cells, and Th17/Th2 subsets, together with heightened expression of immune effector programs [94]. Among FRGs, *CHAC1*, a glutathione-degrading enzyme that promotes ferroptosis, emerges as a central node, exhibiting strong correlations with neutrophil abundance and activated T cell compartments. Mechanistically, *CHAC1*-mediated GSH depletion may act at two levels: in keratinocytes, it lowers the threshold for lipid peroxidation and ferroptotic stress; in immune cells, it may modulate apoptosis and effector function, thereby sustaining inflammatory persistence [93]. From a translational perspective, ferroptosis-based stratification offers several advantages over purely cytokine-centric models. First, it captures upstream metabolic vulnerability rather than downstream immune consequence. Two patients may share comparable *IL-17* signatures yet differing substantially in their epidermal redox state, lipid architecture, and ferroptotic readiness, features that may influence chronicity, flare propensity, and response durability [66,92]. Second, ferroptosis signatures may help explain why a subset of patients remains refractory or only partially responsive to therapies: immune blockade may suppress effector cytokines, but if epidermal membranes remain PUFA-enriched, *GPX4*-deficient, and iron-loaded, the tissue retains the capacity to regenerate inflammatory cues [64]. The key ferroptosis-related genes in psoriasis are summarized in Table 1.

Overall, ferroptosis signatures provide a metabolic lens on psoriasis heterogeneity. However, these stratification models are based on bulk transcriptomic datasets that have not yet been validated in clinical cohorts and, thus, their diagnostic or stratification value remains preliminary.

## 6. Therapeutic Opportunities in Targeting Ferroptosis in Psoriasis

### 6.1. Targeting Ferroptosis Vulnerability in Psoriatic Epidermis

Recognizing that immune dysregulation in psoriasis intersects with epidermal redox and metabolic vulnerability has important therapeutic implications. First, among the “true” ferroptosis modulators, Fer-1 and liproxstatin-1, provide proof-of-principle that, by interrupting lipid peroxidation, psoriasiform inflammation, hyperplasia, and scaling can be attenuated in vivo [19,95]. At present, these data are confined to preclinical settings, and Fer-1 or liproxstatin-1 are not suitable for clinical use. Second, reshaping epidermal iron flux represents a complementary strategy [96]. Local iron chelation, modulation of TFRC activity, or interference with ferritinophagy modulate the epidermis specific iron signaling and blunt catalytic redox pressure while preserving systemic iron homeostasis [97]. Third, boosting cysteine availability, stabilizing GPX4 protein, or modulating upstream regulators such as NRF2 and mechanistic target of rapamycin kinase (mTORC1) improve lipid repair that defines ferroptotic vulnerability [98]. Nutritional and endocrine modulators already used in dermatology, such as vitamin D and selenium, are mechanistically linked to GPX4 expression and activity and may exert part of their benefit by restoring epidermal redox competence [99,100,101]. Furthermore, combining immune suppression with ferroptosis-targeted agents could improve depth and durability of response, reduce flare frequency, and potentially enable dose reduction of immunosuppressive agents, such as anti-IL-17 and anti-TNF [102]. However, targeting ferroptosis in a chronic inflammatory disease demands careful consideration of physiological trade-offs. First, ferroptosis participates in antimicrobial defense and inflammatory resolution in certain contexts [103]. Excessive suppression of lipid peroxidation could blunt innate immune sensing and compromise cutaneous defense against pathogens [104]. Second, ferroptosis is a recognized tumor-suppressive mechanism [105]. Chronic inhibition of ferroptotic pathways could, in principle, reduce elimination of premalignant keratinocytes or alter immune-mediated tumor surveillance. This concern is particularly relevant in a tissue with high turnover and cumulative mutational burden [43]. Epidermis-restricted delivery, temporal modulation, or strategies that reinforce repair rather than block execution may mitigate this risk. Third, keratinocyte differentiation involves tightly orchestrated cell death-like programs [106,107]. Interfering indiscriminately with ferroptosis could perturb terminal differentiation, cornification, or barrier renewal.

### 6.2. Natural Ferroptosis Modulators: Phytochemicals as Pleiotropic Therapeutic Candidates

In addition to pharmacological interventions, several recent studies have highlighted the potential of plant-derived bioactive compounds to modulate redox–metabolic imbalance in psoriasis. Phytochemicals are generally associated with a more favorable safety profile and have long been recognized for their anti-inflammatory and antioxidant properties. Over the past two years, several studies have reported plant extracts with promising activity in preclinical and in vitro models [108], and a few early clinical studies have yielded encouraging results [109]. Anti-psoriatic phytochemicals span a wide range of chemical classes, including flavonoids, phenolic acids, stilbenes, terpenoids, saponins and alkaloids. Despite this diversity, many consistently reduce oxidative stress by stimulating endogenous antioxidant systems such as superoxide dismutase II (SOD2) and catalase, and attenuate inflammation through multiple mechanisms, including inhibition of NF-κB and janus kinase (JAK)/signal transducer and activator of transcription (STAT) signaling, activation of peroxisome proliferator-activated receptor (PPAR) and AMP-activated protein kinase (AMPK) pathways, and suppression of cytokines such as TNF-α and IL-17 [110]. Increasing evidence indicates that their beneficial actions involve additional regulatory dimensions such as ferroptosis, autophagy and epigenetic modulation. Several flavonoids, including quercetin and baicalin, attenuate psoriasiform inflammation in murine and keratinocyte models by restoring GPX4 activity, reducing lipid peroxidation and normalizing iron homeostasis [18,111]. Likewise, diterpenoid and triterpenoid compounds such as andrographolide and ursolic acid ameliorate psoriasis-like lesions while modulating NRF2/heme oxygenase-1 (HO-1) signaling and downregulating ferroptosis-related markers [112,113]. Polyphenol-rich extracts from *Camellia sinensis* and *Curcuma longa* improve histological and molecular features of psoriasis models in parallel with reduced *ACSL4* expression and decreased accumulation of OxPLs [114,115,116]. Given that more than 40 individual phytochemicals and at least 35 plant extracts have already demonstrated anti-inflammatory activity in psoriasis models, the number capable of inhibiting ferroptosis is likely to grow. This expectation is supported by substantial overlap between compounds known to regulate ferroptosis in other disease contexts and those with established anti-psoriatic properties [25,64,117]. Examples include polyphenols and phenolic acids such as curcumin [118], dihydromyricetin [119], apigenin, luteolin, catechins, kaempferol, gallic acid and ellagic acid [108,110], many of which also modulate autophagy [120] and chromatin structure [121], broadening their mechanistic relevance.

Together, these findings suggest that ferroptosis modulation represents an underappreciated component of the biological activity of several plant-derived compounds with anti-psoriatic potential. Nonetheless, clinical evidence for their use in psoriasis is still limited, and both dosing and formulation vary considerably. Their long-term safety also remains unclear. For these reasons, phytochemicals should be seen as promising but still preliminary options, and not as therapies that specifically target ferroptosis.

Table 2 provides an overview of the key therapeutic drug categories designed to target ferroptosis vulnerability in psoriasis.

## 7. Discussion

Collectively, in this review, we suggest that psoriasis can be viewed as a disorder in which redox imbalance, metabolic stress, and immune dysregulation interact. Within this scenario, ferroptosis may represent a key pathogenic mechanism linking keratinocyte vulnerability to chronic inflammation. Keratinocytes undergoing ferroptotic stress indeed release oxidized lipids and reactive aldehydes that can act as danger signals, promote eicosanoid production, and amplify IL-17/TNF/IFN-γ-driven inflammatory circuits through activation of myeloid cells and T lymphocytes [23,25,64]. In parallel, ferroptosis-related transcriptomic signatures reveal a stratifiable metabolic dimension of psoriasis heterogeneity, identifying “ferroptosis-high” endotypes with distinct immune environment and therapeutic liabilities beyond cytokine profiling [66,92,93,94]. From a translational perspective, restoring membrane redox balance, by limiting lipid peroxidation, modulating iron flux, or strengthening GPX4-dependent repair, may complement existing immunotherapies by targeting upstream epidermal sources of inflammation [19,96,97,98]. In this context, several plant-derived bioactive compounds may modulate these redox–metabolic pathways, suggesting an additional strategy to enhance epidermal resilience [108,110,115,117].

## 8. Future Perspectives

Understanding how ferroptosis contributes to psoriasis opens new perspectives for next-generation precision dermatology. However, several questions remain currently unsolved. First, it is still unclear under which conditions ferroptotic stress promotes inflammation or, alternatively, restrains keratinocyte hyperproliferation. Clarifying how factors such as timing, intensity of lipid peroxidation, and local microenvironmental signals influence these outcomes will be important to better understand the role of ferroptosis in the epidermis. Second, current evidence is limited by the low cellular resolution of available datasets. Single-cell and spatial approaches will be essential to identify the specific cell populations in which ferroptosis occurs and to determine how ferroptosis-related signals integrate with local inflammatory circuits. Third, reliable biomarkers of ferroptosis in psoriasis are still lacking. Although several transcriptomic signatures suggest that ferroptosis contributes to disease heterogeneity, these findings remain preliminary and require validation in well-characterized clinical cohorts. Determining whether ferroptosis-related markers can predict disease activity, relapse risk, or treatment response represents an important translational objective. Finally, the therapeutic potential of targeting ferroptosis needs careful evaluation. Experimental inhibitors and phytochemicals have provided important mechanistic insights, but their clinical applicability, specificity, and long-term safety remain uncertain. Future studies integrating genetic models, pharmacological tools, and patient-based analyses will be required to determine whether ferroptosis can be safely and effectively targeted in psoriasis.

## Figures and Tables

**Table 1:** Key ferroptosis-related genes in psoriasis and their enrichment in ferroptosis endotypes.

Gene	Core Function	Role in Ferroptosis	Expression Pattern in Psoriasis and Endotype Assignment
*GPX4* [18]	GSH-dependent detoxification of phospholipid peroxides	Central anti-ferroptotic enzyme maintaining membrane integrity	Decreased in lesions: high-ferroptosis endotype
*ACSL4* [19]	PUFA activation and incorporation into phospholipids	Generates oxidizable PUFA-PLs that promote lipid peroxidation	Increased in lesions: high-ferroptosis endotype
*CHAC1* [93]	Intracellular GSH degradation	Enhances lipid peroxidation by reducing GSH availability	Increased in lesions: high-ferroptosis endotype
*PEBP1* [66]	Modulator of phosphatidylethanolamine oxidation	Facilitates formation of pro-ferroptotic oxidized PE species	Increased in lesions: high-ferroptosis endotype
*CISD1* [66]	Mitochondrial iron sulfur and ROS regulation	Modulates mitochondrial contribution to ferroptotic stress	Increased in lesions: high-ferroptosis endotype
*ACSF2* [66]	Fatty-acid activation and lipid metabolism	Shapes pools of peroxidizable lipids	Increased in lesions: high-ferroptosis endotype
*PRKAA2* (*AMPKα2*) [66]	Metabolic and stress-response regulation	Influences redox homeostasis and ferroptotic susceptibility	Increased in lesions: high-ferroptosis endotype
*GCLC* [66]	Rate-limiting enzyme for glutathione synthesis	Supports antioxidant capacity and GPX4 activity	Increased in lesions: low-ferroptosis endotype

**Table 2:** Therapeutic drug classes targeting ferroptosis vulnerability in psoriasis.

Drug Class	Representative Agents	Evidence in Psoriasis/Ferroptosis	Potential Advantages	Limitations/Considerations
Lipid peroxidation inhibitors [19]	Fer-1	Reduce epidermal hyperplasia, scaling, oxidative stress, and inflammatory cytokines in psoriasiform models	High specificity for ferroptosis; potential for topical or localized delivery.	Risk of suppressing physiological oxidative signaling essential for antimicrobial defense.
	Liproxstatin-1			
Iron chelators/iron flux modulators [97]	Topical chelators	Alleviate redox pressure and normalize iron handling in keratinocytes	Local administration may minimize systemic iron perturbation.	Excessive iron restriction could impair cutaneous innate immunity.
	TFRC modulators			
	ferritinophagy inhibitors			
GPX4 stabilizers and redox repair enhancers [98]	GPX4 stabilizers	Improve redox buffering capacity and reduce ferroptotic sensitivity in psoriatic epidermis	Directly addresses a core driver of ferroptotic vulnerability; may synergize with biologics.	Must avoid interfering with physiological keratinocyte differentiation and cornification.
	GSH precursors			
	cysteine boosters			
	NRF2/mTORC1 modulators			
Combined cytokine blockade + ferroptosis modulation [102]	IL-17 inhibitors	May enhance response durability and reduce relapse by correcting persistent metabolic vulnerability	Potential dose-sparing effect for immunosuppressants.	Excessive ferroptosis inhibition could impair antimicrobial defense or tumor surveillance.
	TNF inhibitors + agents from classes above			
Natural ferroptosis modulators (phytochemicals) [114,115,116,117,118,119]	Quercetin, Baicalin, Andrographolide, Ursolic acid, Curcumin, Catechins	Improve redox homeostasis, reduce inflammatory markers, and modulate ferroptosis-related pathways in preclinical models.	Generally favorable safety profile; broad mechanistic spectrum.	Variable bioavailability and standardization; limited clinical data.

## Data Availability

No new data were created or analyzed in this study. Data sharing is not applicable to this article.

## Abbreviations

The following abbreviations are used in this manuscript:

IL-23	Interleukin-23
IL-17	Interleukin-17
TNF	Tumor necrosis factor
DAMPs	Danger-associated molecular patterns
GPX4	Glutathione peroxidase 4
IMQ	Imiquimod
PUFA	Polyunsaturated fatty acid
MLKL	Mixed Lineage Kinase Domain-Like pseudokinase
TFRC	Transferrin receptor
FTH1	Ferritin heavy chain
FTL	Ferritin light chain
FPN	Ferroportin
ACSL4	Acyl-CoA synthetase long-chain family member 4
LPCAT3	Lysophosphatidylcholine acyltransferase 3
GSH	Glutathione
SLC7A11	Solute carrier family 7 member 11
SLC3A2	Solute carrier family 3 member 2
FSP1	Ferroptosis suppressor protein 1
CoQ10	Coenzyme Q10
GCH1	GTP Cyclohydrolase 1
BH4	Tetrahydrobiopterin
ESCRT-III	Endosomal sorting complex required for transport (ESCRT) complex III
UV	Ultraviolet
ROS	Reactive oxygen species
4-HNE	4-Hydroxynonenal
OxPLs	Oxidized phospholipids
PTGS2	Prostaglandin endoperoxide synthase 2
COX-2	Cyclooxygenase-2
Fer-1	Ferrostatin-1
KRT6	Keratin 6
PASI	Psoriasis Area Severity Index
RSL3	RAS-selective lethal 3
FLG	Filaggrin
NRF2	Nuclear factor erythroid 2-related factor 2
OxPE	Oxidized phosphatidylethanolamine
DE-FRGs	Differentially expressed ferroptosis-related genes
NLR	Nod-like receptor
TLR	Toll-like receptor
RLR	Retinoic acid-inducible gene-I (RIG-I)-like receptor
NCOA4	Nuclear Receptor Coactivator 4
HMGB1	High Mobility Group Box 1
mtDNA	Mitochondrial DNA
IL-33	Interleukin-33
ATP	Adenosine Triphosphate
LL-37	Cathelicidin antimicrobial peptide LL-37
CXCL1/8	C-X-C motif chemokine ligand 1
CCL20	C-C motif chemokine ligand 20
PEBP1	Phosphatidylethanolamine binding protein 1
PRKAA2	Protein Kinase AMP-Activated catalytic subunit alpha 2
ACSF2	Acyl-CoA synthetase family member 2
IFN-γ	Interferon-gamma
NF-κB	Nuclear factor kappa B
GCLC	Glutamate-Cysteine ligase catalytic subunit
CHAC1	ChaC Glutathione-Specific Gamma-Glutamylcyclotransferase 1
CISD1	CDGSH Iron Sulfur Domain 1
TIMM9	Translocase Of Inner Mitochondrial Membrane 9
TRIB2	Tribbles Pseudokinase 2
MDM2	Mouse Double Minute 2
mTORC1	Mechanistic Target of Rapamycin Kinase
SOD2	Superoxide Dismutase II
JAK	Janus Kinase
STAT	Signal Transducer and Activator of Transcription
PPAR	Peroxisome Proliferator-Activated Receptor
AMPK	AMP-activated Protein Kinase
HO-1	Heme Oxygenase-1
LIP	Labile iron pool
